# Notes

**Published:** 1899-08-05

**Authors:** 


### The Institutional Workshop.

### NOTES.

Last Sunday evening a fire occurred in the Salvation Army Maternity Hospital at Hackney, which might easily have led to serious consequences. The outbreak, which was caused by a gust of wind wafting a window curtain against a gas flame, originated in the nursing ward, where several children were lying. For a time several of these little ones were in considerable peril, and one of the nurses was severely burned on the hands and face in her efforts to extinguish the flames. The children were rescued by a policeman, and on the arrival of the brigade the fire was overcome without much difficulty.

Inflammable hangings which blow about are always a danger. Only about a fortnight ago there was a blaze of much the same kind as the above in the infants' pavilion of the Bellevue Hospital in New York. The canopy over a bed caught fire from a candle carried by a nurse, and the flames ran from crib to crib. Fortunately an ambulance driver and several nurses succeeded in pulling down the burning netting, and no great harm was done; but these things show what risks are run when hangings wave about, as they are apt to do when every window is opened to catch any chance breath of fresh air.

A very serious fire is also reported from the Glack Lunatic Asylum, in Aberdeenshire, in which there were at the time about 200 lunatics. The whole building was practically gutted, but fortunately there was time for the inmates to be removed in safety.

While on the subject of fires it may be well to point out that at a fire last week at a building used as a casual pauper test house, at Manchester, not only was the ordinary stone staircase made impassable by smoke, but the supplementary iron staircase was enveloped in flames, so that it was of no service as an escape, and several of the inmates on the third and fourth storeys, being thus cut off, had to jump on to the life sheets held by the firemen. No lives were lost, but some of the inmates, who were burned as well as otherwise injured, had to be taken to the Ancoats Hospital.

London has at last got its municipalities, and it now remains to be seen what it will do with them. The Act seems to contain nothing which is likely to improve the sanitary administration of the metropolis, and indeed the whole question centres on whether it will not make it worse. What seems to us perfectly patent is that so far as sanitary administration is concerned London can only be effectually dealt with by regarding it as one great city, and it remains to be seen whether by raising the status of the municipal boroughs to a higher level and giving them a greater independence than was possessed by the vestries—which is the very essence of the measure—the difficulty of carrying out large sanitary measures and of ensuring uniformity of sanitary administration over large areas will not have been rendered still more difficult than it was before.

Mr. Guntrip King has been appointed secretary to the Samaritan Free Hospital, in succession to Mr. George Scudamore, who, owing to a prolonged illness, has been obliged to resign the post he has held for 27 years.

The late Mr. Brandreth, formerly of Liverpool, has bequeathed £1,000 to Guy's Hospital; £500 to the East London Hospital for Children, the Seaman's Hospital, St. Mary's (Middlesex), and the London Fever Hospital; £200 to the Royal Free Hospital; and £100 each to the Stanley Hospital (Bootle), the Liverpool Royal Infirmary, and the Liverpool Southern Infirmary.

The *West London Medical Journal* very properly draws attention to the serious addition to the work of the West London Hospital which is likely to be involved in the erection just over the way of a huge "doss house," which is to accommodate 800 inmates. This building is being put up by the Rowton Houses (Limited), and the beds are to be let at 6d. per night. As the *West London Journal* suggests, to be within a stone's throw of medical aid such as this hospital affords cannot fail to be regarded as a distinct advantage to its inhabitants.

The Chelsea Hospital for Women has been closed to in-patients to the end of September in order to permit of the following necessary alterations and improvements being carried out: Enlargement and modernising of operating theatre, new hot-water service, new lift, installation of electric light. These improvements will cost nearly £3,000, and, in view of the great pressure at which the work of the hospital is carried on, they can be no longer delayed. An appeal is being made for assistance towards defraying this expenditure. The out-patient department will be closed during August only.

Last week an event of some interest took place at the Hall of the Cordwainers' Company, Cannon Street, when the retiring master, Dr. Alex. Marsden, made a presentation to the company of the handsome service of silver plate publicly subscribed for and presented by H.R.H. the late Duke of Cambridge to his father, Dr. William Marsden, in commemoration of his founding the Royal Free Hospital in 1828. The story of the circumstances under which Dr. Marsden's attention was first directed to the difficulty of gaining admission to hospitals for destitute cases in emergencies is well known, and led to the establishment of the first entirely "free" hospital in London, the London General Institution for the Gratuitous Cure of Malignant Disease, afterwards to become so widely known under its present name. Dr. William Marsden also founded upon similar lines the cancer hospital at Brompton, taking the keenest interest in both charities up to the last day of his life. In 1849 Dr. Marsden was himself master of the Worshipful Company of Cordwainers, and the commemoration plate which bears his name now finds a fitting home amongst its treasures. The service consists of a tall centre-piece, two wine-coolers and four dishes, on the former being a bas-relief of the parable of the Good Samaritan, with a suitable inscription.

